# Beneath the surface: a case report on nonencapsulated *Streptococcus pneumoniae*-associated invasive disease in an immunocompromised patient

**DOI:** 10.1099/acmi.0.000743.v3

**Published:** 2024-02-13

**Authors:** Jonathan Zintgraff, Nahuel Sanchez Eluchans, Paula Gagetti, Celeste Martinez, Dina Pedersen, María Moscoloni, Adrian Lewis, Claudia Lara, Alejandra Corso

**Affiliations:** ^1^​ Servicio de Bacteriología Clínica, Departamento de Bacteriología, INEI-ANLIS "Dr. Carlos G. Malbrán", Ciudad Autónoma de Buenos Aires, Argentina; ^2^​ Servicio Antimicrobianos, Departamento de Bacteriología, INEI-ANLIS "Dr. Carlos G. Malbrán", Buenos Aires, Argentina; ^3^​ Servicio de Bacteriología, Hospital Municipal de Agudos Dr. Leónidas Lucero, Bahía Blanca, Provincia de Buenos Aires, Argentina; ^4^​ Servicio de Microscopia Electrónica, ANLIS "Dr. Carlos G. Malbrán", Buenos Aires, Argentina

**Keywords:** nonencapsulated *Streptococcus pneumoniae*, WGS, invasive pneumococcal disease, transmission electron microscopy

## Abstract

**Background.:**

*Streptococcus pneumoniae*, a prominent human pathogen linked to various systemic diseases, includes non-typeable pneumococci marked by the absence of a detectable capsule. However, the majority of invasive infections are attributed to encapsulated strains. This case report details the first documented instance of invasive disease caused by non-typeable *S. pneumoniae* in Argentina since 2017.

**Case Presentation.:**

A 19-year-old woman presented with haemorrhagic injuries attributed to chronic oral mucosa irritation. Subsequent hospitalization revealed bone marrow aplasia, leading to antibiotic, antifungal, antiviral, and immunosuppressive treatments, culminating in her discharge. Two weeks later, she was readmitted with sepsis related to a respiratory focus, exhibiting a negative COVID-PCR test. After ten days, ICU admission revealed additional infections: positive COVID-PCR test, fungal sinusitis, and *S. pneumoniae* bacteremia. Targeted treatments led to improvement, and the patient was subsequently discharged.

**S. pneumoniae characterization.:**

Verification of the capsule’s absence utilized traditional methods such as the Quellung reaction, transmission electron microscopy, molecular assays, and Whole Genome Sequencing (WGS). The isolate, identified as ST18335, displayed genetic features and antibiotic resistance patterns, concordant between WGS and the agar dilution method. It demonstrated non-susceptibility to penicillin and cefotaxime, based on meningitis breakpoints, as well as meropenem and cotrimoxazole.

**Conclusion.:**

This case underscores the clinical significance of non-typeable *S. pneumoniae*, emphasizing the necessity for a comprehensive approach to identification and characterization. The findings contribute to ongoing discussions regarding the challenges posed by non-typeable strains in vaccine development, understanding clinical impacts, and addressing antibiotic resistance. As the pneumococcal epidemiological landscape evolves, this case serves as a valuable addition to the evolving knowledge surrounding non-typeable *S. pneumoniae*, highlighting the continued need for surveillance and research in infectious diseases.

## Data Summary

The whole-genome sequence data generated for this study are available in the European Nucleotide Archive (ENA), and the accession number is ERR12137906.

## Introduction

*Streptococcus pneumoniae*, commonly referred to as pneumococcus*,* holds global significance as a human pathogen, playing a pivotal role in the occurrence of systemic diseases [1, 2]. Culture-based methods for identification typically rely on characteristics such as morphology, bile solubility, optochin susceptibility, and the Quellung reaction [3]. However, exceptions exist, including optochin-resistant pneumococci [4, 5], bile-insoluble strains [6], and those lacking a specific Quellung reaction agglutination due to the absence of a capsule [7, 8]. This latter group is commonly referred to as non-typeable (NT) pneumococci and is frequently encountered in colonization [9]. However, NT strains are seen infrequently in diseases such as conjunctivitis (including large outbreaks) [10, 11], acute otitis media [12], acute exacerbations in patients with chronic obstructive pulmonary disease [13], and more recently, invasive diseases [14].

We described the first NT *S. pneumoniae* causing invasive pneumococcal disease in Argentina since 2017, with a full profile study of this isolate, involving three different approaches: traditional characterization, molecular characterization and Whole Genome Sequencing (WGS).

## Case report

A 19-year-old woman with a medical history of delayed maturation and epilepsy was brought to the emergency room (ER) at Hospital Municipal de Agudos Dr. Leónidas Lucero in Bahía Blanca, Buenos Aires, Argentina. On admission she had multiple haemorrhagic injuries of twelve hours of evolution, due to a chronic irritation in the oral mucosa known as ‘Morsicatio buccarum’. Laboratory tests revealed a classic pancytopenia, evidenced by haemoglobin 9.4 g dl^−1^, hematocrit 28%, white blood cell counts 0.70 mm^−3^ and platelets 9.000 mm^−3^. Results for tests detecting human immunodeficiency virus, hepatitis viruses, herpes simplex viruses (HSV-1 and HSV-2), cytomegalovirus (CMV), syphilis, and enterovirus were all negative.

The patient was admitted to the hospital and had a bone marrow examination that revealed a severe bone marrow aplasia. Considering the patient’s compromised immune system and clinical presentation, the medical team in the emergency room opted to initiate treatment with cephazolin, gentamicin, acyclovir, nystatin, and fluconazole. Five days later, the patient started immunosuppressive treatment involving cyclosporine and thymoglobulin. An allogeneic transplant was requested, but the major histocompatibility (MHC) complex compatibility test performed on her two brothers turned out to be not compatible. Four days later the patient was discharged.

Two weeks later, the patient was readmitted to the ER due to a sepsis associated to a respiratory focus, and empiric treatment with ceftazidime and amikacin was initiated. She was transfused with red cells and platelets and COVID-PCR test was negative.

**Fig. 1.**
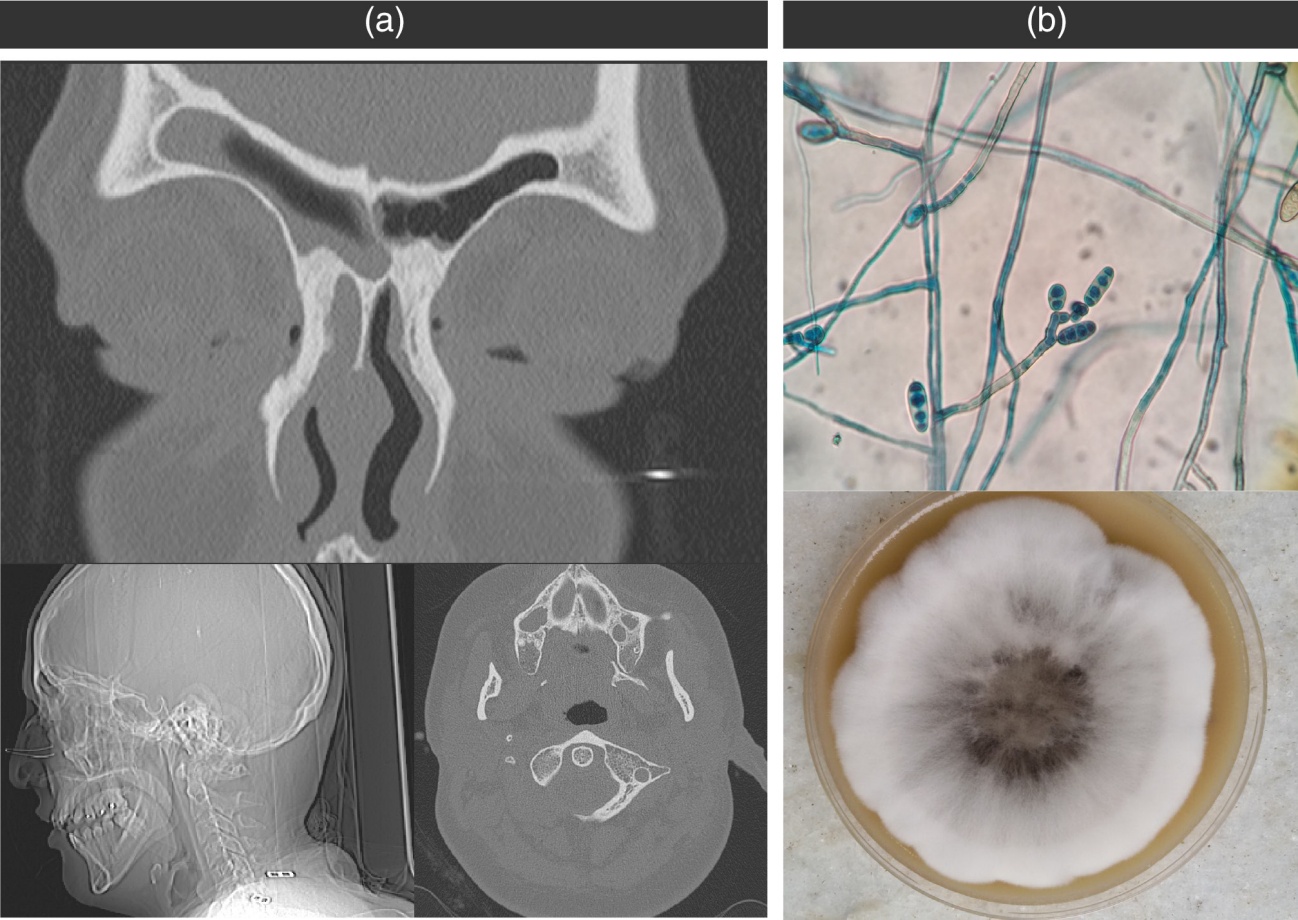
(a) The Computerized Axial Tomography (CAT) scan exhibited a consolidation in the right maxillary sinus and notable thickening of the mucosa in both the left maxillary and sphenoid sinuses. (**b)** Microscopic examination of *Bipolaris* spp. microculture (lactophenol blue stain, 400×) and microculture on sabouraud agar.

Day 10, the patient was admitted to the Intensive Care Unit and several microbiological cultures were performed, included urine culture and carbapenem-resistant Enterobacterales (CRE) perianal swabs surveillance culture, all of which were negative. Two days later, the patient started with odynophagia and productive cough, a COVID-PCR was performed, and it was positive. After 5 days, the patient refers right cervical pain with mastoid involvement and otalgia. A computerized axial tomography (CAT) scan disclosed complete consolidation in the right maxillary sinus and partial involvement of the ethmoidal sinuses (Fig. 1a). A sample was collected to investigate a suspected opportunistic infection, revealing the presence of septate hyphae (Fig. 1b). Furthermore, the diagnosis of sinusitis associated with phaeohyphomycosis was confirmed and treatment with voriconazole was implemented.

Before initiation of antimicrobial treatment, she was blood cultured with a positive result within 24 h. The Gram-stain revealed Gram-positive diplococcus and empirical treatment with vancomycin and ampicillin sulbactam was started. Preliminary cultures yielded *S. pneumoniae,* and the isolate was forwarded to the National Reference Laboratory (NRL) to confirm the identification and perform capsular serotyping and antimicrobial susceptibility tests.

The patient’s condition improved gradually and 15 days later she was discharged home.

At the present, she is being followed as an outpatient in the general clinics.

The National Reference Laboratory (NRL) verified that isolate 25 041 was indeed *S. pneumoniae* through conventional assessments, including optochin susceptibility and bile solubility [15].

### Isolate characterization

The Quellung reaction is considered the gold standard method for serotyping [16]. With 100 capsular serotypes identified for *S. pneumoniae* [17], this technique employs a high-quality microscope and specific pneumococcal antisera (Statens Serum Institut – Copenhagen, Denmark). The procedure entails testing a pneumococcal cell suspension with both pooled and specific antisera targeting the capsular polysaccharide. Microscopic observation is used to detect antigen-antibody reactions. A positive Quellung reaction indicates the binding of pneumococcal capsular polysaccharide with type-specific antibodies found in the typing antiserum.

While optochin susceptibility, bile solubility, and Quellung testing serve as the established standards for the identification and serotyping of pneumococci, atypical pneumococci often yield inconsistent results [18]. Non-typeable *S. pneumoniae* refers to isolates that fail identification using capsular polysaccharide typing sera. These non-typeable pneumococci can be categorized into isolates lacking capsule genes, those possessing capsule genes but displaying a phenotypic lack of encapsulation, and those exhibiting a phenotypic resemblance to *S. pneumoniae* but with genetic distinctions from typical pneumococci [19].

**Fig. 2.**
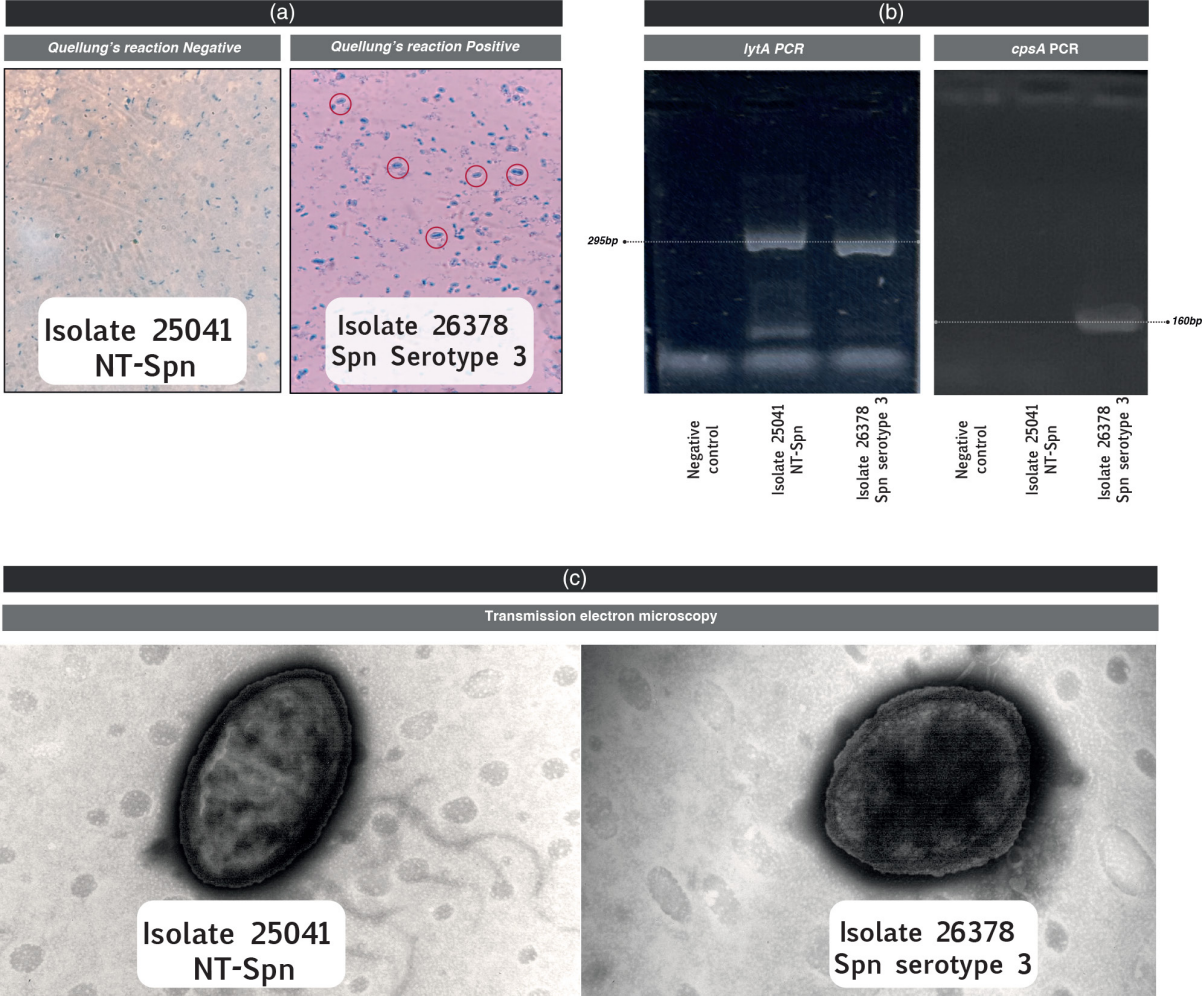
(a) Quellung’s reaction: isolate 25 041, classified as NT-Spn, exhibited no reactivity in any of the anti-serum tests. In contrast, isolate 26 378, identified as *S. pneumoniae* serotype 3 (positive control), yielded a positive reaction with the specific anti-serum. (**b)** Gel electrophoresis of *S. pneumoniae*-specific PCR products targeting the 295 bp *lytA* gene. Lane 1: negative control; lane 2: isolate 25041 NT-Spn; lane 3: isolate 26 378 *S*. *pneumoniae* serotype 3. And 160 bp *cpsA* gene Lane 1: negative control; lane 2: isolate 25041 NT-Spn; lane 3: isolate 26 378 *S*. *pneumoniae* serotype 3. (**c)** Negative stain (phosphotungstic acid). On the left the Isolate 25041 NT-Spn demonstrates loss of the capsule compared with the image on the right, isolate 26 378 *S*. *pneumoniae* serotype 3, with the polysaccharide capsule surrounding the cell of the pneumococcal isolate.

In our case, the isolate did not react with any of the pools described (Fig. 2a), therefore in order to realize a full analysis of the isolate, a molecular approach was applied. Confirmation of *S. pneumoniae* identification was performed through several specific assays. Firstly, a conventional PCR targeting the *lytA* gene [20] was conducted, yielding a positive result (Fig. 2a). The positive *lytA* assay, along with the characteristic responses in optochin susceptibility and bile solubility (as mentioned before), collectively confirmed the accurate identification of the isolate as *S. pneumoniae*.

Early genetic investigations [21] revealed the tight linkage of *S. pneumoniae* genes essential for the biosynthesis and expression of capsular polysaccharide (CPS) to the pneumococcal chromosome. In order to validate the existence of capsular polysaccharide in our isolate, we performed a *cpsA* PCR [22], specifically targeting a highly conserved gene present in all capsular loci. The result was negative (Fig. 2b).

It is important to underscore that a negative *cpsA* result does not definitively indicate a non-serotypable isolate. Absence of the *cpsA* band has been observed during PCR serotyping of some isolates, mainly serotypes 25 and 38 [23], and occasionally serotypes 14 [24] and 35A. Therefore, caution should be exercised when interpreting the absence of *cpsA* amplification as an indication of non-serotypeability. In light of this, we proceeded with Whole Genome Sequencing (WGS) to further investigate whether the isolate belonged to a NT-Spn or if a new serotype was involved.

### Principle of the Quellung reaction

The Quellung reaction operates on the principle of anticapsular antibodies within the serum interacting with the carbohydrate material of the pneumococcal capsule. This interaction induces a microprecipitin reaction on the surface of *S. pneumoniae*. The resultant antigen-antibody reaction causes a refractive index change in the capsule, rendering it visibly ‘swollen’ and distinct.

Following the introduction of a counterstain (methylene blue), pneumococcal cells become dark blue, enclosed by a well-defined halo representing the outer boundary of the capsule (Fig. 2a). The transmitted light through the capsule appears brighter compared to both the pneumococcal cell and the background. Positive Quellung reactions may be observed in single cells, pairs, chains, or clumps of cells.

Upon identifying an isolate as a pneumococcus, sequential testing with antisera pools is conducted until a positive reaction occurs. Each pool of antiserum comprises various mixtures of antisera developed against 100 pneumococcal serotypes. Once the pool is established, individual type and group antisera included in the reactive pool are tested sequentially. Type antisera typically react with a single serotype (e.g. type three antiserum reacts with serotype three), while group antisera react with all serotypes within a specific group (e.g. group 24 antiserum reacts with serotypes 24A, 24B and 24F). Further differentiation of some serotypes within groups is achieved using factor antisera (e.g. serotype 24A reacts with factor 24d, but not 24c or 24e). Testing continues with all relevant factor antisera until the serotype is conclusively determined [25, 26].

### Species identification, multi locus sequence typing (MLST) and and *in silico* serotyping through whole-genome sequencing (WGS) analysis

The strain underwent sequencing at the ‘Unidad Operativa, Centro Nacional de Genómica y Bioinformática, ANLIS Dr. Carlos G. Malbrán’ using the Nextera XT DNA Sample Prep Kit (Illumina, San Diego, CA, United States) on a MiSeq sequencer, producing 250 bp paired-end reads. The raw data have been submitted to the European Nucleotide Archive (ENA), and the accession number is ERR12137906. The reads quality was evaluated with FastQC. Label taxonomic was carried out with Kraken two, to confirm the species identification [27]. Kraken 2.0 confidently classified approximately 82 % of sequences as *Streptococcus pneumoniae* species, while only 3.28 % were attributed to the *Streptococcus mitis* group*.*

Unicycler Galaxy version 0.4.8.0 was employed for *de novo* assembly, and genome annotation was executed using Prokka Galaxy version 1.14.6. The search for *lytA* and *cpsA* was conducted using JBrowse.

The assembly was uploaded to Pathogenwatch [28] which assigns serotype by Seroba [29], GPSC by PopPUNK [30] and reports the MLST profile and the inferred resistance genotype. The isolate was ST18335 and the results of Pathogenwatch report are shown in Table 1.

**Table 1.** WGS analysis

Method	Result
Genes by WGS	
*IytA*	*Present*
*cpsA*	*Absent*
Kraken 2.0	82 % *S. pneumoniae*
SeroBA	Swiss_NT
PopPUNK	GPSC not assigned
MLST	ST18335

### Transmission electron microscopy

Negative staining with phosphotungstic acid was employed to visualize and characterize the ultrastructure of the isolate [31]. Briefly, carbon-coated copper grids were first treated with a solution 1 % (w/v) of Alcian Blue to enhance hydrophilicity. A small drop of the sample inactivated by incubation with 2 % paraformaldehyde in buffer for 30 min at 25 °C followed by an additional 30 min at 37 °C was then applied to the grid surface and left to adsorb for ten minutes. Excess liquid was gently blotted with filter paper, avoiding grid damage. Subsequently, a 2 % aqueous solution of phosphotungstic acid was applied as the negative stain for 30 s. The excess stain was carefully removed, and the grid was left to air-dry at room temperature. Then, the specimens were examined under a transmission electron microscope at an appropriate accelerating voltage. Negative staining with phosphotungstic acid provided high contrast images, enabling the visualization of fine structural details of the samples at the nanometer scale (Fig. 2c).

### Antimicrobial analysis

We were intrigued by the potential role of NT-Spn as reservoirs of antibiotic resistance, prompting us to assess the resistance profiles of the strains against a panel of 14 antibiotics.

Antimicrobial susceptibility testing was conducted using the agar dilution method for penicillin, amoxicillin, cefotaxime, meropenem, ceftaroline, ceftobiprole, erythromycin, tetracycline, doxycycline, chloramphenicol, cotrimoxazole, levofloxacin, rifampicin, and vancomycin, following CLSI guidelines [32, 33]. Penicillin resistance was defined as a minimum inhibitory concentration (MIC) of ≥0.12 µg ml^−1^, in accordance with the meningitis breakpoint. For cefotaxime, intermediate and resistant categories were designated at MIC values of 1 and ≥2 µg ml^−1^, respectively. Ceftobiprole interpretation followed EUCAST breakpoints: S (susceptible) ≤0.5 µg ml^−1^; R (resistant) ≥1 µg ml^−1^. Strains of *S. pneumoniae* ATCC 49619 and *Staphylococcus aureus* ATCC 29213 served as quality control organisms.

Table 2 displays the minimum inhibitory concentrations (MICs) obtained through the agar dilution method as well as MICs to β-lactam agents inferred from WGS according to the PBP (Penicillin-Binding Protein) profile, which in this isolate was PBP1a=34, PBP2b=16, and PBP2x=new. Resistance to cotrimoxazole was inferred by *folP* indels and *folA* I100L and susceptibility to the other antibiotics by the absence of specific genes. A complete correlation was observed in all the cases.

**Table 2.** Susceptibility profile by agar dilution method and inferred from WGS data

	Reference agar dillution method		WGS analysis	
Antimicrobial agent	MIC (µg ml^−1^)	Interpretation	Inferred MIC (µg ml^−1^)	Interpretation
Amoxicillin (Meningitis breakpoint)	1.0	S	2.0	S
Penicillin (Meningitis breakpoint)	1.0	R	2.0	R
Cefotaxime (Meningitis breakpoint)	1.0	I	1.0	I
Ceftaroline	0.12	S	na	na
Ceftobiprole	0.25	S	na	na
Meropenem	0.5	I	0.5	I
Cotrimoxazole	8.0	R	na	R
Erythromycin	0.06	S	na	S
Tetracycline	1.0	S	na	S
Doxycycline	0.25	S	na	S
Levofloxacin	2.0	S	na	S
Chloramphenicol	2.0	S	na	S
Rifampicin	0.03	S	na	na
Vancomicin	0.5	S	na	na

I, intermediate; NA, not available; R, resistant; S, susceptible.

In summary, the examined isolate showcased resistance to penicillin and cotrimoxazole, while demonstrating intermediate resistance to cefotaxime. Conversely, susceptibility was observed in relation to the other antibiotics subjected to testing.

## Discussion

*S. pneumoniae* stands as a noteworthy human pathogen, responsible for various infectious diseases, including pneumonia, otitis media, meningitis, and septicemia. It has traditionally been categorized into serotypes based on capsule diversity. However, some isolates lack a detectable capsule, making them non-typeable (NT-Spn). The emergence of NT-Spn poses concerns for vaccine strategies, pathogenicity, and antibiotic resistance patterns.

A notable rarity in this case was a bloodstream infection caused by NT-Spn. The capsule is believed crucial for evading immunological recognition and rapid clearance from the bloodstream [34].

While the phenomenon of NT-Spn was reported decades ago [35], recent advancements in molecular typing techniques have brought it renewed attention. NT strains have been observed in carriage studies and disease scenarios, underlining their clinical relevance. The absence of a capsule in these strains could be due to the loss or alteration of the capsule locus, typically located in the pneumococcal genome, or the presence of novel capsule types unaccounted for by current typing methods.

The emergence of NT-Spn prompts critical questions. Firstly, the clinical implications of NT strains need thorough investigation. Studies by Keller *et al*. suggest NT pneumococci may be less susceptible to immune recognition [36], potentially enhancing their ability to evade host defences. This could impact disease outcomes, transmission dynamics, and colonization. Additionally, the effectiveness of current pneumococcal vaccines against NT strains is unclear, as these vaccines target specific capsule types. Understanding if NT strains contribute to vaccine failures is vital for vaccine development and implementation.

The rising prevalence of antibiotic resistance among pneumococcal isolates has raised concerns about the potential association between NT *S. pneumoniae* and antibiotic resistance [37]. However, the resistance profile of the isolate studied did not differ from *S. pneumoniae* isolated from invasive disease in Argentina.

Addressing knowledge gaps regarding NT *S. pneumoniae* requires collaborative efforts among researchers, clinicians, and public health agencies. Comprehensive genomic studies are needed to elucidate the genetic mechanisms behind capsule loss and identify potential virulence factors unique to NT strains [38]. Moreover, extensive epidemiological investigations are essential to ascertain the prevalence and clinical significance of non-typeable pneumococci across diverse settings.

Our analysis demonstrated consistent *lytA* presence and *cpsA* absence between conventional PCR and WGS. These findings affirm the accurate identification of our strain as *Streptococcus pneumoniae*. However, SeroBA and PopPUNK did not yield predictions regarding serotype or GPSC (https://www.pneumogen.net/gps/assigningGPSCs.html), respectively. Regarding the MLST profile, the PubMLST website did not yield an exact match and a novel ST18335 was assigned. The closest ST identified was ST1156, a double locus variant (DLV) described in three non-typeable isolates from Portugal nearly twenty years ago [39].

Summarizing, we present a clinical case of an immunocompromised patient who experienced a COVID-19 infection along with two opportunistic infections: a fungal rhinosinusitis and an invasive infection by a non-typable *S. pneumoniae* strain. From a clinical perspective, it is crucial to acknowledge the potential limitations of the initial treatment for oral mucosal irritation. While the initial administration of cefazolin, gentamicin, acyclovir, nystatin, and fluconazole may appear broad in addressing oral mucosal issues, the laboratory results revealing the patient’s immunocompromised condition and the complex clinical presentation necessitated a more comprehensive approach. This approach was implemented by the medical team in the emergency room, assuming that they considered mucosal irritation as a potential infectious focus within the context of the patient’s compromised immune system. The goal of this approach is to effectively address potential bacterial, fungal, and viral infections associated with her bone marrow aplasia.

Moving forward, from a microbiological point of view, we successfully confirmed the identity of the strain and the absence of its polysaccharide capsule through a combination of phenotypic, molecular tests, transmission electron microscopy, and whole-genome analysis. This case illustrates the value of whole-genome analysis for a strain that could not be serotyped using the Quellung reaction, allowing us to determine it was a non-capsulated strain rather than a new serotype or a serotype that was not expressing its capsule. Non-typeable *S. pneumoniae* is an emerging topic in infectious diseases. The increasing recognition of NT strains emphasizes the need for comprehensive research to understand their clinical significance, impact on vaccine strategies, and antibiotic resistance patterns. With the evolving pneumococcal epidemiology, vigilant monitoring of NT *S. pneumoniae* is crucial for developing effective public health interventions against pneumococcal infections.
